# FALL PREVENTION PROGRAMS FOR RURAL COMMUNITY DWELLING OLDER ADULTS RESULTS IN IMPROVED BALANCE

**DOI:** 10.1093/geroni/igz038.3149

**Published:** 2019-11-08

**Authors:** Robin E McAtee, Ronni Chernoff, Kathryn Packard, Whitney Thomasson, Laura Spradley, Cynthia Mercado

**Affiliations:** 1 University of Arkansas for Medical Sciences, Little Rock, Arkansas, United States; 2 Univ Arkansas for Medical Sciences, Little Rock, Arkansas, United States; 3 UAMS, Hot Springs, Arkansas, United States

## Abstract

More than one in four older adults (OA) in the US fall each year and 20 to 30% suffer moderate to severe injuries such as head trauma or hip fractures. Falls are both a medical and financial burden that can be significantly reduced by lifestyle interventions. One of the main strategies to help reduce these statistics is evidence-based fall prevention classes which help to build muscle, improve balance, and increase participants’ confidence in fall control. A goal of the Arkansas Geriatric Workforce Enhancement Program (HRSA grant U1QHP28723) is to deliver community-based programs that improve health outcomes and quality of life. Evidence-based preventive health training is a major way to meet this goal. Two of these evidence-based trainings are Tai Chi (TC) and A Matter of Balance (AMOB), multi-week classes that help to improve balance and reduce fall risk. This study took place at scheduled community-based classes, where demographic and balance data were obtained. The aim was to determine the difference in the balance of community-dwelling OA as measured by Balance Tracking System® before and after AMOB and TC courses. Both groups showed positive changes in their mean balance percentage with the AMOB class having a higher Mean ± SD (24.3 ± 21.0) vs. the TC participants (4.0 ± 29.7). The One-Way Analysis of Variance showed statistically significant difference in the AMOB class over those in the TC class, F (1, 32) = 5.280, p < 0.05. The Cohen’s d = 0.789 indicates a large effect between the two groups.

